# The Impact of Phenotypic and Genotypic G6PD Deficiency on Risk of *Plasmodium vivax* Infection: A Case-Control Study amongst Afghan Refugees in Pakistan

**DOI:** 10.1371/journal.pmed.1000283

**Published:** 2010-05-25

**Authors:** Toby Leslie, Marnie Briceño, Ismail Mayan, Nasir Mohammed, Eveline Klinkenberg, Carol Hopkins Sibley, Christopher J. M. Whitty, Mark Rowland

**Affiliations:** 1 Department of Infectious and Tropical Diseases, London School of Hygiene & Tropical Medicine, London, United Kingdom; 2 HeathNet TPO, Peshawar, North West Frontier Province, Pakistan; 3 Department of Genome Sciences, University of Washington, Seattle, Washington, United States of America; 4 HealthNet TPO, Amsterdam, The Netherlands; University of Melbourne, Australia

## Abstract

**Background:**

The most common form of malaria outside Africa, *Plasmodium vivax,* is more difficult to control than *P. falciparum* because of the latent liver hypnozoite stage, which causes multiple relapses and provides an infectious reservoir. The African (A−) G6PD (glucose-6-phosphate dehydrogenase) deficiency confers partial protection against severe *P. falciparum*. Recent evidence suggests that the deficiency also confers protection against *P. vivax,* which could explain its wide geographical distribution in human populations. The deficiency has a potentially serious interaction with antirelapse therapies (8-aminoquinolines such as primaquine). If the level of protection was sufficient, antirelapse therapy could become more widely available. We therefore tested the hypothesis that G6PD deficiency is protective against vivax malaria infection.

**Methods and Findings:**

A case-control study design was used amongst Afghan refugees in Pakistan. The frequency of phenotypic and genotypic G6PD deficiency in individuals with vivax malaria was compared against controls who had not had malaria in the previous two years. Phenotypic G6PD deficiency was less common amongst cases than controls (cases: 4/372 [1.1%] versus controls 42/743 [5.7%]; adjusted odds ratio [AOR] 0.18 [95% confidence interval (CI) 0.06–0.52], *p* = 0.001). Genetic analysis demonstrated that the G6PD deficiency allele identified (Mediterranean type) was associated with protection in hemizygous deficient males (AOR = 0.12 [95% CI 0.02–0.92], *p* = 0.041). The deficiency was also protective in females carrying the deficiency gene as heterozygotes or homozygotes (pooled AOR = 0.37 [95% CI 0.15–0.94], *p* = 0.037).

**Conclusions:**

G6PD deficiency (Mediterranean type) conferred significant protection against vivax malaria infection in this population whether measured by phenotype or genotype, indicating a possible evolutionary role for vivax malaria in the selective retention of the G6PD deficiency trait in human populations. Further work is required on the genotypic protection associated with other types of G6PD deficiency and on developing simple point-of-care technologies to detect it before administering antirelapse therapy.

*Please see later in the article for the Editors' Summary*

## Abstract 2

Analyses of a case-control study among Afghan refugees in Pakistan find that a G6PD (glucose-6-phosphate dehydrogenase) “Mediterranean” type deficiency confers substantial protection against *Plasmodium vivax* malaria.

## Editors' Summary

**Background:**

Malaria is a parasitic infection transmitted to people through the bite of an infected mosquito. Although *Plasmodium falciparum* is responsible for most malaria deaths, *P. vivax* is the commonest, most widespread cause of malaria outside sub-Saharan Africa. Like other malaria parasites, *P. vivax* has a complex life cycle. Infected mosquitoes inject a parasitic form known as sporozoites into people where they replicate inside liver cells without causing symptoms. About 8–9 days later, merozoites (another parasitic form) are released from the liver cells and invade red blood cells. Here, they replicate rapidly before bursting out and infecting more red blood cells. This increase in the parasitic burden causes malaria's characteristic symptoms (debilitating and recurring chills and fevers). *P. vivax* infections are usually treated with chloroquine (although resistance to this drug is now emerging) but patients must also take primaquine, a drug that kills hypnozoites, a form of *P. vivax* that hibernates in the liver. Hypnozoites can cause a relapse months after the initial bout of malaria and make *P. vivax* malaria harder to control than *P. faciparum* malaria.

**Why Was This Study Done?:**

Some mutations (DNA changes) protect their human carriers against specific disease-causing organisms. These mutations occur at high frequencies in populations where these organisms are common. For example, the widespread distribution of mutations that cause a deficiency in an enzyme called glucose-6-phosphate dehydrogenase (G6PD) mirrors the distribution of malaria and the African (A−) form of G6PD deficiency, a type of G6PD deficiency that is common in people of African origin, is known to provide partial protection against severe *P. falciparum* malaria—*P. falciparum* does not thrive in G6PD-deficient red blood cells. In areas where *P. vivax* malaria is common, Mediterranean and Asian variants of G6PD deficiency are more widespread than A− G6PD, so the question is, do these variants protect against *P. vivax* malaria? In this case-control study (a study in which the characteristics of people with and without a specific condition are compared), the researchers investigate whether G6PD deficiency protects against *P. vivax* infection in a population of Afghan refugees living in Pakistan.

**What Did the Researchers Do and Find?:**

The researchers enrolled 372 Afghan refugees who had had *P. vivax* malaria during the previous two years and 743 refugees who had not had malaria over the same period. They measured G6PD activity in the participants' blood to detect “phenotypic” G6PD deficiency (reduced enzyme activity) and looked for the Mediterranean variant of the G6PD gene in the participants (“genotypic” G6PD deficiency). 5.7% of the controls but only 1.1% of the cases had phenotypic G6PD deficiency. Statistical analyses indicated that participants with reduced G6PD levels were about one-fifth as likely to develop *P. vivax* malaria as those with normal G6PD levels after allowing for other factors that might affect their susceptibility to malaria, an adjusted odds ratio (AOR) of 0.18. The genetic analysis indicated that the Mediterranean G6PD gene variant provided protection against *P. vivax* infection in men (AOR 0.12) and in women carrying either one or two defective copies of the G6PD gene (AOR 0.37); because the G6PD gene is on the X chromosome, men have only one copy of the gene but women have two copies.

**What Do These Findings Mean?:**

These findings indicate that Mediterranean-type G6PD deficiency protects against *P. vivax* malaria infection in this population of Afghan refugees. Although further studies are needed to determine whether other G6PD variants protect against *P. vivax* malaria, these findings suggest that *P. vivax* malaria might be responsible for the retention of the G6PD deficiency trait in some human populations. In addition, these findings may have implications for the treatment of *P. vivax* malaria. Currently, in most places where *P. vivax* malaria is common, primaquine is not given routinely because primaquine can trigger red blood cell death (hemolytic anemia) in G6PD-deficient people and tests for G6PD deficiency are rarely available. These findings suggest that the risk of exposure to primaquine among people infected with *P. vivax* might be lower than previously assumed, because G6PD deficiency is less common among *P. vivax*-infected patients than among the general population. Nevertheless, these findings are unlikely to increase the use of primaquine immediately. Such an increase, the researchers suggest, will only occur if a simple test for G6PD deficiency is developed.

**Additional Information:**

Please access these Web sites via the online version of this summary at http://dx.doi.org/10.1371/journal.pmed.1000283.

- Information is available from the World Health Organization on malaria (in several languages)
- The US Centers for Disease Control and Prevention provides information on malaria (in English and Spanish)
- Information is available from the Wellcome Trust on all aspects of malaria, including a news item about G6PD deficiency protecting against severe P. falciparum malaria
- MedlinePlus provides links to additional information on malaria (in English and Spanish)
- The Malaria Vaccine Initiative has a fact sheet on Plasmodium vivax malaria
- Vivaxmalaria provides information about *P. vivax*
- More about G6PD deficiency can be found on KidsHealth from the Nemours Children's Health System

## Introduction

The majority of malaria outside sub-Saharan Africa is caused by *Plasmodium vivax,* which causes up to 390 million clinical cases a year amongst a population at risk of approximately 2.6 billion [1],[2]. Its wide geographical distribution is due, in part, to its ability to undergo development (sporogony) in mosquitoes at a lower temperature than *P. falciparum* and to its formation of a latent liver stage (the hypnozoite), which initiates secondary blood-stage infections [3]. These characteristics make vivax malaria significantly more difficult to control or eliminate than falciparum malaria.

Glucose-6-phosphate dehydrogenase (G6PD) deficiency is the most common genetic enzymopathy in humans with more than 200 variants identified to date [4]. Because the trait is X-linked and recessive, expression of phenotypic deficiency occurs more frequently in males than females. G6PD deficiency is relatively rare in populations where malaria is rare but may exceed 10% in prevalence where malaria is common [5]. In patients with G6PD deficiency, treatment with 8-aminoquinoline drugs such as primaquine may cause methemoglinaemia [6] and precipitate haemolytic anaemia which, although often mild and subclinical, can be severe in some people with particular G6PD deficiency variants [7],[8]. The potentially life-threatening interaction between the enzyme deficiency and anti-hypnozoite drugs places a major constraint on the ability of endemic countries to control or eliminate vivax malaria, because G6PD testing is not available in most areas with vivax malaria, so safe treatment of hypnozoite infections with 8-aminoquinoline drugs is not deemed feasible [9].

The concordance of the distribution of G6PD deficiency with maps of historical malaria led to the hypothesis that the trait is protective against *P. falciparum*. This hypothesis was confirmed in endemic areas of Africa where the African (A−) variant of G6PD deficiency protected against severe disease [10]–[12]. Falciparum malaria is now thought to be the principal evolutionary driver for retention of the A− variant of G6PD deficiency [10]–[12]. Mediterranean and Asian variants of G6PD deficiency are, however, more common in areas where vivax malaria is endemic. Mediterranean variants tend to cause more significant haemolysis than the A− variant when challenged with antimalarial drugs, fava beans, or other environmental stressors. A recent study [13] demonstrated that one Asian variant (G6PD-Mahidol) was associated with lower levels of parasitaemia in Thai adults with vivax malaria, but not with *P. vivax* infection or with *P. falciparum*. Genetic evidence for an evolutionary interaction was also demonstrated. Vivax malaria, often incorrectly assumed to be benign [14],[15], seems likely to exert evolutionary pressure for retention of the G6PD trait in human populations. We therefore investigated whether G6PD deficiency is protective against vivax malaria infection in a South Asian population in Pakistan.

## Methods

Using a case-control design, we examined the association of G6PD deficiency with vivax malaria, retrospectively, over 2 years of observation. The study was conducted in three Afghan refugee villages in North West Frontier Province, Pakistan (Adizai, Baghicha, and Kaghan). The refugee villages had populations ranging from 1,000 to 3,500 people. Malaria transmission was seasonal and hypoendemic [16],[17] with the majority (approximately 95%) of cases being due to vivax malaria and the remainder to *P. falciparum*. Ethnically, the population is Pashtun [18].

### Selection of Cases and Controls

In November and December 2006, cases were selected from residents of the refugee villages who in earlier studies were diagnosed with vivax malaria [19],[20]. Cases were defined as individuals who had had at least one episode of vivax malaria in the previous 2 years based on research slides collected using standard methods and double read by two experienced microscopists. Discordant slides were read by a third microscopist. A cross-checking procedure was in place in all clinics and maintained accuracy at >98% [21] (HealthNet TPO, unpublished data, 2004–5). In addition, PCR was conducted on blood samples taken from cases during one of the studies [19] (*n* = 30), which confirmed that all tested cases were indeed infected with *P. vivax*.

Village health records were used to select community controls from the same population as cases. Controls had no known episodes of malaria during the same 2 years of observation. The record system contained information on every family, and each family had a health card on which every member was recorded by name. The registration was regularly updated for births and deaths during consultation in the village health clinics. If a malaria blood smear was taken, the slide result was recorded against the patient's record (even if it was negative). If the patient was parasitaemic, the treatment given was also recorded. The records are maintained by trained village health workers and clinic staff. This allowed the compilation of a list of all individuals in the villages who had not had malaria over the 2 years before the study. All residents from the same villages as cases who were more than 2 years old and had no history of malaria in their clinic record were therefore eligible for enrolment as controls.

Controls were selected at random from the complete list of eligible village residents. A bottle was spun outside the house of each case in order to select a random house and if it contained an eligible control (of the same sex and within 5 years of age of the case), then the control was enrolled. If no suitable control was available, the bottle was re-spun until a suitable control was found from a house in the vicinity of the case. The selection was therefore controlled for the likely confounding factors of age and village (associated with risk of infection) and sex (associated with risk of G6PD deficiency) to ensure that disease and exposure risk was broadly similar between cases and controls. Once informed consent was given, the control was assessed by microscopy for current malaria parasitaemia, and if positive the control was not included. Because no controls had parasitaemia at the time of the survey, none were excluded on the basis of this criterion. Information on treatment-seeking practice was collected to identify those individuals who had sought medical care outside the village in the last 2 years; a restricted analysis, in which such individuals were excluded, was conducted as a check against potential bias resulting from malaria that was not recorded in clinic records. Children under the age of 2 years were excluded since they would not have been born at the start of the observation period.

Blood samples were collected in EDTA tubes and immediately tested for phenotypic G6PD deficiency using a colorimetric test (Sigma Diagnostics, Poole, Dorset, UK) according to the manufacturer's instructions. Samples found to be deficient were tested a second time. Each participant gave a blood sample stored on filter paper for genetic analysis. The filter papers were allowed to dry, marked with the participant's identifier and stored in individual bags. The samples selected for analysis were sent to the Department of Genome Sciences at the University of Washington, Seattle, USA.

### Ethics Statement

Ethical approval was obtained from the London School of Hygiene and Tropical Medicine Ethics Review Board and from the Pakistan Medical Research Council Committee on Bioethics. All participants gave written informed consent to participate. For the gene sequencing, ethical approval was given by the University of Washington Ethics Committee.

### Statistical Analysis

The primary analysis examined the association between phenotypic G6PD deficiency and vivax malaria. A sample size was calculated for a 2∶1 ratio of controls to cases. A sample of 500 cases and 1,000 controls gave 80% power to detect a frequency of phenotypic G6PD deficiency of 1% amongst cases and 3.5% amongst controls [19],[20] at the 95% confidence level.

We conducted univariate and multivariate analysis using unconditional logistic regression and adjusting for prespecified potential confounding factors between cases and controls; those factors included in the final unconditional multivariate logistic regression model for the primary outcome were age group, sex, tribe, and refugee village. In response to suggestions from the statistical referee we undertook an additional post-hoc analysis using conditional logistic regression, which was not part of the prespecified analytical plan. In this analysis each case was matched with the two controls identified in their village by bottle-spinning and was thus matched for village, sex, and age group. We included tribe as a potential confounding factor in the model.

G6PD deficiency is an X-linked mutation so the secondary analysis examined the association between the deficiency (both phenotypic and genotypic) and infection separately for males and females. This analysis took the same approach as before and applied unconditional logistic regression and a post-hoc conditional logistic regression. The genotyped samples were used to assess the level of protection conferred by confirmed hemizygous deficiency in males and heterozygous and homozygous deficiency in females. The study was not powered specifically for the secondary analyses. Data was analysed using STATA v8 (STATA Corp, College Station, TX, USA).

### Genetic Analysis

Participant blood samples were examined for G6PD genotype in two phases. Initially, we selected half of the samples from males who were phenotypically deficient and the requisite number of male non-deficient samples (in an approximate 1∶2 ratio). We then amplified a 5.2 kb fragment using the primers 13125F: 5′ GTT TAT GTC TTC TGG GTC AGG GAT GG 3′ and 18396R: 5′ AGT GTT GCT GGA AGT CAT CTT GGG T 3′ from Saunders et al, [22]. Forty-six out of 57 of the samples that we attempted to amplify gave good sequence across the majority of that fragment (up to exon 10) and showed that the predominant allele was the Mediterranean (Med) type.

In the second phase we conducted PCR amplification of Med allele fragments on the 40% sample of the participants whose samples were available, in equal proportion between cases and controls. These comprised the same 1∶2 ratio of cases to controls and allowed examination of the frequency of the Med− allele between cases and controls. Med allele fragments were amplified in a 20 µl reaction volume containing: 1× PCR PreMix A, 0.2 µM each primer, 0.5 units FailSafe enzyme mix (Epicentre, Madison, WI), and 2 µL of template DNA. Cycling conditions were: 94°C for 3 min; 36 cycles of 94°C for 30 s, 55°C for 30 s, 62°C for 60 s; 62°C for 5 min, 15°C for 5 min. Sequencing was done in a 10 µl volume, using 1–3 µl of PCR product, 1.7 µl 5× sequencing buffer for Big Dye (400 mM Tris-HCl, 10 mM MgCl_2_, pH = 9.0), 0.8 µM primer, and 1 µl Big Dye (Applied Biosystems, Foster City, CA). Products of sequencing reactions were separated on an ABI 3130xl (Applied Biosystems, Foster City, CA) and analyzed using Sequencher (Gene Codes Corporation, Ann Arbor, MI). The following primers were used for the Med allele: Medf: 5′ ATGATGCAGCTGTGATCCTCACTC 3′ and Medr: 5′ ATGAGGTTCTGCACCATCTCCTTG 3′.

## Results

A total of 372 cases and 743 controls were enrolled into the study at a ratio of roughly 1∶2. Table 1 shows enrolment data for cases and controls. There were no differences in the frequency of sex, village, or age group between cases and controls, although tribal group did differ. There was a lower than anticipated number of participants, because some of the cases (identified in the earlier studies [19],[20]; *n* = 125) had either repatriated to Afghanistan or had been enrolled more than once in the previous studies (but were included only once in the present survey). All participants were ethnic Pashtuns.

**Table 1:** Characteristics of cases and controls.

Variable	Cases (*n* = 372)	Controls (*n* = 743)
**Mean age (y)**	13.6	13.5
**Age group, ** ***n*** ** (%)** a		
2–10	213 (57.2)	395 (53.4)
11–20	104 (28.0)	237 (32.0)
>20	55 (14.8)	108 (14.6)
**Sex, % male**	42.2	42.7
**Ethnic group (Pashtun, %)**	100	100
**Tribe, ** ***n*** ** (%)** b		
Safi	167 (44.9)	346 (46.4)
Mamund	39 (10.5)	126 (17.0)
Shinwari	29 (7.8)	47 (6.3)
Tarakheil	23 (6.2)	32 (4.3)
Unknown	67 (18.0)	175 (23.5)
Other	47 (12.6)	17 (2.3)
**Refugee village, ** ***n*** ** (%)**		
Adizai	288 (77.4)	575 (77.4)
Baghicha	65 (17.5)	130 (17.5)
Kaghan	19 (5.1)	38 (5.1)

a Data missing from four controls.

b Chi^2^ = 59.5, *p*<0.001.

There was a lower frequency of phenotypic G6PD deficiency amongst cases (4/372 [1.1%]) than controls (42/743 [5.7%]), Chi^2^ = 13.1, *p*<0.001) (Table 2). Unconditional univariate analysis was conducted to identify prespecified potential confounding associations with G6PD deficiency. The prespecified variables entered into the unconditional logistic regression model were sex, age group, refugee village, and tribe (Table 2). The primary analysis demonstrated significant protection in those with phenotypic G6PD deficiency with adjusted odds ratio (AOR) 0.18 [95%CI 0.06–0.52], *p* = 0.001. Overall, tribe was associated with the deficiency, and there was some variation between individual tribal groups (Table 2). For the post-hoc matched analysis, the AOR (0.17 [95% CI 0.06–0.48], *p* = 0.001) was similar to the unmatched analysis (Table 2) although the association with tribe was not seen.

**Table 2:** Effect of malaria, sex, age group, village, and tribe on frequency of phenotypic G6PD.

Variable	G6PD Deficient, *n/N* (%)	Odds Ratio (95% CI)	P value	Adjusted Odds Ratio (95% CI)	P Value
**Malaria history**					
Control	42/743 (5.7)	1		1	
Case	4/372 (1.1)	0.18 (0.06–0.51)	0.001	0.18 (0.06–0.52)	0.001
**Sex**					
Male	33/473 (7.0)	1		1	
Female	13/641 (2.0)	0.28 (0.14–0.53)	<0.001	0.30 (0.15–0.59)	0.001
**Age group (y)**			0.59		0.93
2–10	26/608 (4.3)	1		1	
11–20	15/341 (4.4)	1.03 (0.54–1.97)	0.9	0.93 (0.46–1.86)	0.8
>20	5/163 (3.1)	0.71 (0.27–1.87)	0.5	0.95 (0.33–2.71)	0.9
**Refugee village**			0.03		0.004
Adizai	27/863 (3.1)	1		1	
Baghicha	14/195 (7.2)	2.39 (1.23–4.66)	0.01	2.48 (1.01–6.04)	0.046
Kaghan	5/57 (8.8)	2.98 (1.10–8.04)	0.03	3.05 (0.98–9.46)	0.054
**Tribe**			0.02		0.007
Safi	25/513 (4.8)	1		1	
Mamund	7/165 (4.2)	0.86 (0.37–2.04)	0.7	0.57 (0.23–1.45)	0.2
Shinwari	8/76 (10.5)	2.30 (1.00–5.30)	0.051	0.93 (0.31–2.82)	0.9
Tarakheil	2/55 (3.6)	0.74 (0.17–3.20)	0.7	1.04 (0.23–4.69)	1.0
Unknown	4/242 (1.7)	0.33 (0.11–0.95)	0.04	0.22 (0.07–0.67)	0.008
Other	0/64	—	—	—	—

Because the G6PD gene is located on the X chromosome, sex-stratified analysis was also conducted. The frequency of deficiency in males (7.0%) was higher than for females (2.0%). Amongst males, G6PD deficiency was less likely in cases than in controls (AOR 0.12 [95%CI 0.03–0.49], *p* = 0.004). Amongst females we did not detect evidence of an effect (AOR 0.35 [95%CI 0.08–1.61], *p* = 0.2), the number of deficient females being too low to make a robust estimate (*n* = 2/215) (Table 3). For the post-hoc matched analysis, the AOR was again similar between the unmatched analysis (Table 3) and the matched analysis (AOR from matched analysis, amongst males: 0.11 [95% CI 0.03–0.51], *p* = 0.004 and amongst females: 0.35 [95% CI 0.07–1.67, *p* = 0.19).

**Table 3:** Unadjusted ORs and AORs for phenotypic G6PD deficiency stratified by sex.

	*n/N* (%)	OR (95% CI)	*p*-Value	AOR^a^ (95% CI)	*p*-Value
**Males**					
Control	31/316 (9.8)	1		1	
Case	2/157 (1.3)	0.12 (0.03–0.50)	0.004	0.12 (0.03–0.49)	0.004
**Females**					
Control	11/426 (2.6)	1		1	
Case	2/215 (0.9)	0.35 (0.08–1.61)	0.2	0.35 (0.08–1.61)	0.2

a Odds ratios adjusted for village (unpublished data); age group and tribe were dropped due to no univariate effect (age group) or low sample sizes (tribe).

A total of 418 samples were successfully sequenced to provide genotype data (Table 4). As expected, all of the hemizygous G6PD-deficient males (*n* = 16) and homozygous deficient females (*n* = 2) in the sample were phenotypically deficient, and all genotypically G6PD normal males and females were phenotypically nondeficient. Of the heterozygous females 24/32 (75%) were phenotypically normal and 25% were phenotypically deficient. All individuals identified as carrying the mutant allele had a C to T change at nucleotide 563, which is characteristic of the Mediterranean variant and similar to alleles found in India [23] and Iran [24]. Six samples (three male and three female) yielded discordant results showing phenotypic deficiency but a normal genotype. All of these were controls (Table 4). Attempts to examine other possible variants (Orissa, Chatham, and Cosenza) were unsuccessful.

**Table 4:** Sample description for genotyped samples.

	Controls *n/N* (%)	Cases *n/N* (%)
Genotyped	298/743 (40.1)	150/372 (40.3)
Yielding sequence	278/298 (93.3)	140/150 (93.3)
Discordant (males)^a^	3/124 (2.4)	0/62
Discordant (female)^a^	3/154 (2.0)	0/78

a Discordant: Phenotype deficient, but with wild type for the Med- allele. The three discordant females were all homozygous WT against the Med allele assay.

The secondary analysis, conducted using G6PD genotype as the explanatory variable, showed that females carrying a single or double copy of the deficiency were partially protected against vivax malaria infection (pooled AOR: 0.37 [95% CI 0.15–0.94], *p* = 0.037) (Table 5). Amongst heterozygous females, there was weak evidence of protection although the sample size was small for this analysis (Table 5). Only three homozygous deficient females were found, and none of these were cases. In hemizygous deficient males the AOR was 0.12 (95% CI 0.02–0.92), *p* = 0.041.

**Table 5:** Frequency of G6PD deficiency genotype between cases and controls.

	Control *n/N* (%)	Case *n/N* (%)	Univariate		Multivariate^a^	
			OR (95% CI)	*p*- Value	OR (95% CI)	*p*-Value
**Males**						
G6PD normal^b^	109 (87.8)	61 (98.4)	1		1	
Hemizygous deficient	15 (12.1)	1 (1.6)	0.12 (0.02–0.92)	0.042	0.12 (0.02–0.92)	0.041
**Females**						
Homozygous Normal	126 (81.8)	72 (92.2)	1		1	
Heterozygous	26 (16.9)	6 (7.7)	0.40 (0.16–1.03)	0.057	0.40 (0.16–1.02)	0.055
Homozygous deficient	2 (1.3)	0	—	—		
Heterozygous and homozygous	28 (18.2)	6 (7.7)	0.38 (0.15–0.95)	0.038	0.37 (0.15–0.94)	0.037

a Odds ratios adjusted for village (unpublished data); age group and tribe dropped due to nonsignificance (age group) and low sample sizes (tribe).

b Both wild-type and Med variant alleles shared the same basic B allele background as found in GenBank accession no. L44140 and cited in Saunders et al. [22], but we identified three indels, within introns at positions, 584.1, 628.1, and 1450.1 (numbering based on Saunders 5.2 kb fragment). The only difference between the Med variant and wild-type alleles was the single nucleotide change at nt 563.

The frequency of the allele for G6PD deficiency among male controls was 12.1% (15/124), and because males carry a single copy of the X-linked gene, this approximates the population frequency of the deficient allele (Table 5). Among the female controls, there were 16.9% (26/154) heterozygotes and 1.3% (2/154) homozygotes for G6PD deficiency. Therefore, if the frequency of the allele among controls approximates the population genotype frequencies, more than 90% (26/28) of G6PD deficiency alleles in females occur as heterozygotes and only 7.4% (2/28) as homozygotes; this is similar to the genotype frequencies in females predicted by the Hardy-Weinberg model (i.e., 1.5% homozygotes for deficiency, 21.3% heterozygotes, 77.3% G6PD normal) based on a G6PD deficient allele frequency of 12.1% (Chi^2^ = 1.03, *p* = 0.69). Among cases, the frequency of the allele among males (1.6%) and females (7.7%) was much lower, and being protective against malaria these will differ from the gene frequency in the general population.

To examine evidence of possible misclassification of cases as controls, several factors that could have led to misclassification were considered using a restricted analysis against the primary outcome, as follows. Less than one-third of the controls reported using health facilities outside the village as their source of consultation for minor illness or malaria treatment and diagnosis (30.0%, 13.2%, respectively). Also amongst controls, 26/743 (3.5%) had self-reported malaria, which was not recorded in clinic records over the period of observation and 5/743 (0.7%) had ambiguous clinic records. In addition, 202/727 (27.8%) of controls were family members of cases. The restricted analysis, in which all individuals who were in these categories were excluded, produced results that did not differ from the unrestricted analysis.

## Discussion

The study demonstrated that G6PD deficiency, whether measured by phenotype or genotype, is associated with substantial protection against vivax malaria infection. Among heterozygous females the deficiency was associated with protection less often than in hemizygous males. In populations where G6PD deficiency has been measured it rarely occurs at a frequency greater than 15% [4],[5],[9],[11]. Even at this upper limit of the frequency range, the majority of phenotypically G6PD deficient females will be heterozygous. Heterozygous females may be either phenotypically deficient or normal depending on the relative proportion of circulating deficient and nondeficient red cells, because females exhibit mosaicism in expression of G6PD [25]. In our sample, 75% of heterozygous females were phenotypically normal, despite which there was a trend towards protection in this group.

The discovery that G6PD deficiency is protective against *P. vivax* infection is supported by its geographical distribution and may explain why the more severe variants (Mediterranean and Asia) are seen in vivax-predominant areas. This study is supported by recently published findings [13] that the G6PD Mahidol deficiency, an Asian strain, is associated with reduced parasitaemia and had experienced strong selection pressure from about 2,500 years ago. We have demonstrated a stronger protection against infection by the Mediterranean variant. The relationship between the type of G6PD variant and level of protection requires further investigation but the contrary findings presented here and in Thailand suggest that it differs between variants. Too few cases had G6PD deficiency in our sample to reliably analyse its effect on parasite density amongst cases.

Malaria is considered by some to be the single greatest source of selection pressure acting on human populations originating from endemic areas [12],[26], and the geographical distribution of several genetic polymorphisms that are protective against malaria are consistent with this theory [26],[27]. The finding that G6PD deficiency is protective against severe *P. falciparum* malaria in African variant G6PD-deficient children [10]–[12] is one example of this evolutionary interaction. Our findings provide strong empirical evidence, and complement the evolutionary genetic findings of Louicharoen et al. [13], that *P. vivax* has exerted considerable selection pressure on human populations. This pressure could come either through mortality—severe vivax malaria being much more common than previously realised [14],[15]—or through an indirect socioeconomic effect on human fecundity [2],[27]. *P. vivax* is known to cause anaemia and low birthweight in pregnancy [28] and the effect of malaria in young children can lead to mental and physical retardation [2]. A recent evaluation of the clinical picture of vivax malaria has identified complications which had not been previously acknowledged as common, for example, severe anaemia, cerebral malaria, renal failure, hepatic dysfunction, acute respiratory distress syndrome, sepsis and shock [14],[15]. Most areas outside Africa that are endemic for vivax malaria also have falciparum malaria. Recent data describing the relative proportion of the two species is inconsistent with evolutionary time, and drawing conclusions as to the relative impact of these species on human evolution and the retention of the G6PD gene in coendemic areas requires caution. However, vivax malaria is thought to have originated in Asia [29] and to have existed for longer than falciparum [30],[31]. Furthermore, vivax malaria is known to have been endemic to Africa before the emergence between 30,000 and 10,000 years ago of the Duffy negative trait which confers almost complete immunity to *P. vivax* [27], providing indirect evidence that vivax has exerted evolutionary pressure on human populations. Vivax could certainly have contributed to the retention of the deficiency.

The discovered protection is biologically plausible. G6PD-deficient erythrocytes infected by *P. falciparum* are known to be more sensitive to the oxidative stress produced by the parasite [4]. The resulting cellular damage impairs parasite growth, causes lysis of infected cells, and stimulates early phagocytosis of erythrocytes. This is likely to be as relevant in vivax as falciparum malaria. Lysis of G6PD-deficient red cells challenged with oxidative stress is, in part, dependent on the age of the cell. The African deficiency variant generally causes significant lysis only in older erythrocytes, but the Mediterranean variant also causes damage and ultimately lysis of young erythrocytes (reticulocytes) in the presence of a variety of stressors such as drugs or infection. Vivax malaria preferentially infects reticulocytes whilst falciparum malaria infects erythrocytes of all ages [26]. The Mediterranean variant G6PD deficiency is one of the most common of the severe deficiencies and has potential adverse negative selective effects (such as favism). Its wide distribution across Asia may be explained if it confers greater protection against vivax than against falciparum malaria.

Potential shortcomings of the case-control design include misclassification of cases as controls at the selection stage. Misclassification will be limited because asymptomatic infections are rare (none were found in this study) and almost all treatment was given in village clinics. Any such misclassification is unlikely to explain the observed result because any dilution would underestimate the effect of G6PD deficiency on vivax infection. Similarly, misclassification due to patients with *P. falciparum* being wrongly diagnosed as having *P. vivax* is unlikely in the present study because the highly trained research microscopists were routinely subjected to quality control through slide re-examination [21]; secondly, all slides were double-read (or triple-read); thirdly, PCR was conducted on 30 samples from cases which confirmed the microscopic diagnosis; fourthly falciparum malaria, at the time of the study, was very rare in this area.

G6PD deficiency is heritable and therefore could be clustered at the household level and provide a source of sampling bias. However, when family members of cases were excluded from the analysis, the overall conclusions were unchanged; again, residual clustering would lead to an underestimate of the observed effect. Failure of laboratory determination of G6PD phenotypic deficiency is another potential source of error. The high concordance of genotypic and phenotypic deficiency suggests this is not a major factor in this study. It is probable that some controls with discordant results (i.e., normal for the Mediterranean variant but phenotypically deficient) were, in fact genuinely G6PD deficient for other variants that may exist at low levels in this population. The small proportion of discordant phenotypic and genotypic results makes this unlikely to be a significant source of bias in this study.

In case-control studies the association between two factors does not necessarily prove the direction of causality except in the case, as here, of innate genetic conditions. Measured potential confounding factors had little effect on the outcome. We conclude that it is unlikely the apparent substantial protective effect of G6PD deficiency arose by chance or from bias in the study design.

In public health terms, the current treatment practice in most settings where vivax malaria is common is not to use antirelapse therapy in proven cases of vivax malaria because of the risk of haemolysis when testing for phenotypic G6PD deficiency is not available. This restriction is one of the major barriers to effective control and eventual elimination because hypnozoites constitute the reservoir of infection. The findings presented here indicate that, assuming accurate diagnosis, the risk of exposure to primaquine amongst G6PD-deficient individuals with vivax malaria would be lower than previously assumed because the prevalence of G6PD in vivax-infected patients would be significantly lower than in the general population. It is important to examine the level of protection conferred by other types of G6PD variant occurring in other vivax-endemic areas. The risk of severe haemolysis among G6PD-deficient individuals administered with other regimens of primaquine is also unclear; literature on this topic is sparse. For example, in Mediterranean variant G6PD deficiency, haemolysis with primaquine can be severe and, on the basis of limited data, might be quite common if full 14-day courses are taken [32] whereas weekly primaquine for 8 weeks seems safe in Pashtun populations [20]. For a more informed risk analysis, additional data is required, plus modelling to assess the potential effects of G6PD variant, epidemiological background, sex, dose, or duration of treatment on the risk of haemolysis in deficient individuals.

Under prevailing conditions, however, G6PD testing is rarely available and G6PD deficiency goes largely undetected, so our finding is unlikely to increase access to antirelapse drugs in the near future because of the continuing risk of haemolysis. Hence the hypnozoite reservoir will remain unchallenged and will continue to contribute to morbidity through relapse and transmission. For effective control and treatment, either a simple test for G6PD deficiency or an antirelapse drug that can be safely given to patients with G6PD deficiency is required. This study, however, shows that the probability of individuals with vivax malaria being deficient for G6PD may be significantly lower than in the general population from which they are drawn.

## Abbreviations

AOR: adjusted odds ratio
